# Synthesis of novel galactose functionalized gold nanoparticles and its radiosensitizing mechanism

**DOI:** 10.1186/s12951-015-0129-x

**Published:** 2015-10-09

**Authors:** Chuan-dong Zhu, Qin Zheng, Li-xue Wang, Han-Feng Xu, Jin-long Tong, Quan-an Zhang, Yuan Wan, Jian-qing Wu

**Affiliations:** Department of Geriatrics, The First Affiliated Hospital of Nanjing Medical University, 210019 Nanjing, Jiangsu People’s Republic of China; Department of Oncology, The Second Hospital Affiliated of Southeast University, 210003 Nanjing, Jiangsu People’s Republic of China; PMR Institute, 201203 Shanghai, People’s Republic of China; PerMed Biomedical Co. Ltd., 575 Bibo Road, 201203 Shanghai, People’s Republic of China

**Keywords:** Gold nanoparticles, Galactose, Polyethylene glycol, γ-H2AX, Radiosensitization, Hepatocellular carcinoma

## Abstract

**Background:**

Biocompatible gold nanoparticles (GNPs) are potentially practical and efficient agents in cancer radiotherapy applications. In this study, we demonstrated that GNPs can significantly modulate irradiation response of hepatocellular carcinoma cells in vitro and investigated the underlying mechanisms. We co-grafted galactose (GAL) targeting hepatocyte specific asialoglycoprotein receptor and Polyethylene Glycol (PEG) onto GNPs surfaces to increase GNPs targeting specificity and stability.

**Results:**

This novel GAL-PEG-GNPs and bare GNPs show similar appearance and cytotoxicity profiles, while more GAL-PEG-GNPs can be effectively uptaken and could enhance cancer cell killing.

**Conclusion:**

GAL-PEG-GNPs have better radiosensitization to HepG2. The sensitization mechanism of GAL-PEG-GNPs is related to the apoptotic gene process activated by generation of a large amount of free radicals induced by GNPs.

## Background

Radiation therapy plays an increasingly important role in the treatment of hepatocellular carcinoma (HCC), particularly in the management of locally advanced unresectable HCC [1, 2]. However, radiation-induced liver disease and radiation resistance are major factors that limit the success of radiation therapy [3–5]. To improve the effectiveness of radiation therapy, image-guided radiation that provides real-time imaging of the tumor target during treatment has been study. Real-time imaging can help compensate for normal movement of the internal organs from breathing and for changes in tumor size during treatment. Moreover, in contrast to tumor control it enables full therapeutic efficacy while reducing the degree and frequency of invasive interventions [6].

On the other hand, radioprotectors and radiosensitizers, chemicals that modify a cell’s response to radiation are also under development. Radioprotectors are drugs that protect normal cells from damage caused by radiation therapy [7]. In contrast, radiosensitizers make tumor cells more susceptible to radiation damage and can increase the damaging effects of radiation while minimizing exposure to normal and healthy cells [8]. Several radiosensitizers, such as misonidazole, metronidazole, tirapazamine, trans sodium crocetinate are under study [9]. In addition, some anticancer drugs, such as 5-fluorouracil and cisplatin, can make cancer cells more sensitive to radiation therapy as well [10–12]. Together, radiosensitizers could enable radiation therapy at a lower dose, but would not affect treatment efficiency.

Nanoparticles have played a key role in the enhancement of the radiation therapy by acting as both a therapeutic and a carrier for other therapeutics. Particularly, inert and biocompatible gold nanoparticles (GNPs) with tunable size and low osmolality hold many potential advantages [13]. Gold absorbs approximately 3-times more than iodine at 20 and 100 keV; the dose enhancement factor for gold is adjustable depending on the beam’s energy and the amount of gold delivered; the biodistribution of GNPs can be imaged in real time before a therapeutic dose is delivered and used for treatment planning and quantified prediction of dose enhancement; moreover, GNPs allow surface immobilization of antibodies, peptides, aptamers or drugs. Thus, in recent years, using GNPs to enhance the radiation absorbed dose deposited in tumors from X-ray has gained increasing attention [14–17]. Ligands functionalized GNPs could enhance radiation sensitivity in radiation-resistant human prostate cancer cells at keV and MeV X-rays [15]. Chithrani et al. also showed a range of cell line specific responses including decreased clonogenic survival, increased apoptosis and induction of DNA damage could be induced when exposure to 1.9 nm GNPs and X-rays [17].

Ligands against cancer cell surface biomarkers or extracellular matrix proteins have led to the applications of ligands for specific delivery of therapeutic agents [18]. Asialoglycoprotein receptor (ASGPR) is a hepatocyte-specific receptor, and approximately 5 × 10^5^ ASGPRs are expressed on the sinusoidal surface of a single liver cell [19]. ASGPR mediates the capture and endocytosis of galactose- or *N*-acetylgalactosamine-terminating glycoproteins [19, 20]. Thus, galactose, asialofetuin, acetylgalactosamine and asialoorosomucoid targeting ASGPR are commonly used as homing agents for drug delivery [21].

**Fig. 1:**
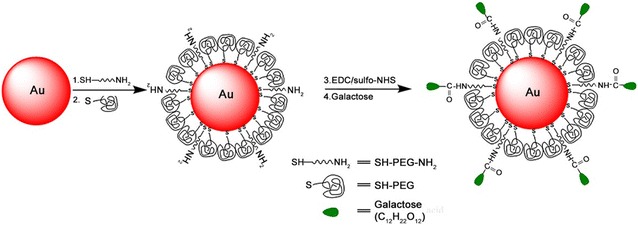
The schematic diagram of preparation of GNPs and GAL-PEG-GNPS. GNPs in 20 nm diameter were prepared using sodium citrate reduction method. To prepare GAL-PEG-GNPs, 0.1 mL of bare GNPs at 0.1 mg/L (pH 7.5) were mixed with excessive amount of thiol-PEG-amino and incubated at 4 °C overnight, followed by thiol-PEG grafting. 100 μL of sulfo-NHS and EDC (5:1, pH 5.0) were incubated with 20 μg of GAL for 6 h at 37 °C to active carboxyl groups of GAL first, and 20 μL of PEGylated GNPs at 0.1 mg/L (pH 7.2) were incubated with the mixture at 37 °C for 2 h to finally produce GAL-PEG-GNPs

We propose that ASGPR-targeted GNPs may enhance the cytotoxic effects of radiation therapy, as well as concentrate the effect on targeted tumor cells in tissue. β-d-galactose (GAL) as a homing agent thus is immobilized onto bare GNPs surfaces. Further, to prevent GNPs from being quickly phagocytosed by reticuloendothelial system (RES) [22], biodegradable and biocompatible polyethylene glycol (PEG) is co-grafted to form a novel composite known as GAL-PEG-GNPs (Fig. 1). Results showed that compared to bare GNPs, GAL-PEG-GNPs can be more quickly and effectively uptaken by ASGPR over-expressed HepG2 cells. HepG2 cells treated by GAL-PEG-GNPS showed significant DNA double-strand breaks and cell apoptosis after 6-MeV X-rays irradiation. These results demonstrated that our GAL-PEG-GNPs hold potential as a radiosensitizer for HCC therapy.

## Results

### Characterization of GAL-PEG-GNPs

**Fig. 2:**
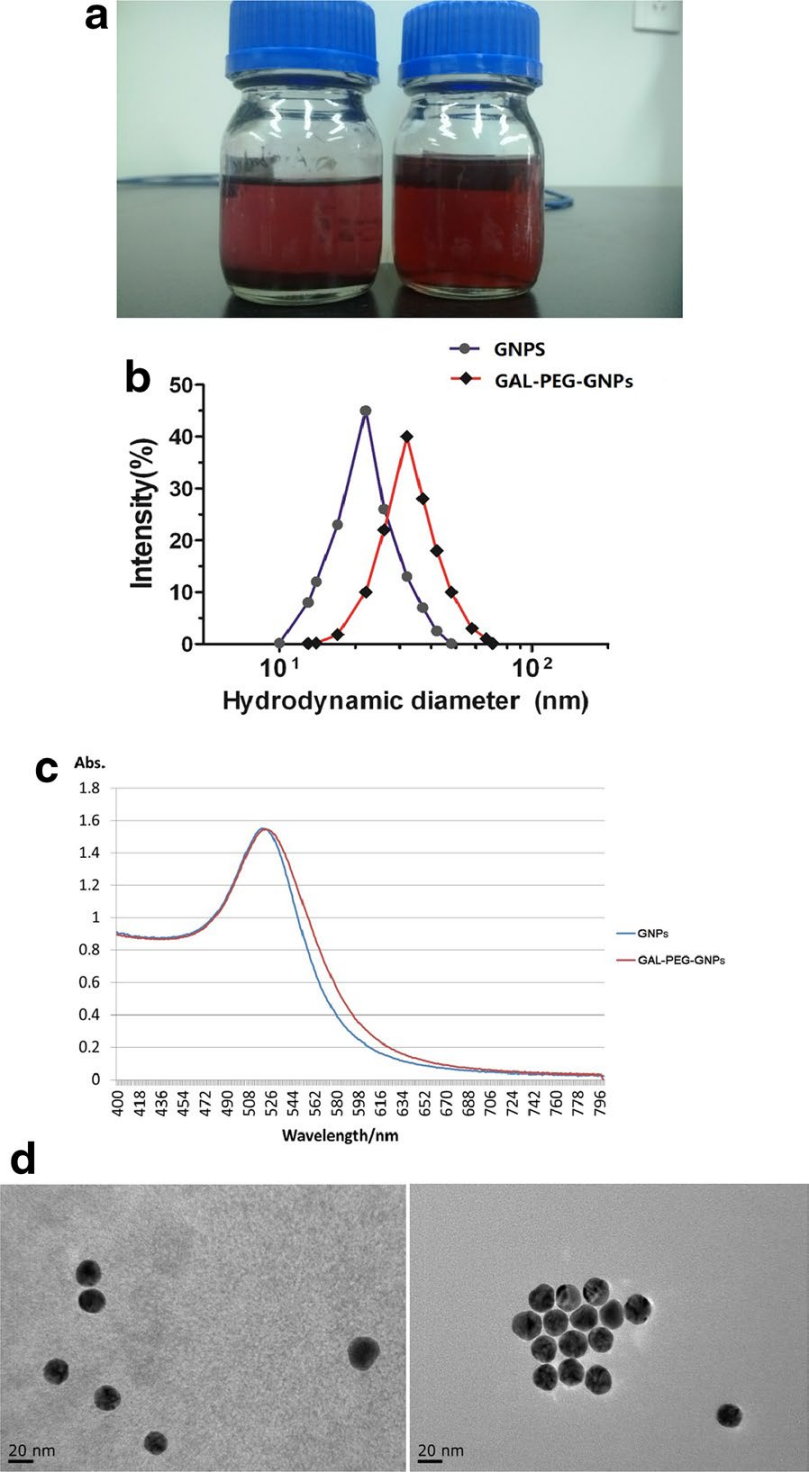
Characterization of nanoparticles. **a** Shows appearance of GNPs (*left*) and GAL-PEG-GNPs (*right*); **b** shows size distribution of GNPs (*blue*) and GAL-PEG-GNPs (*red*), respectively; **c** shows absorption peak of GNPs (*blue*) and GAL-PEG-GNPs (*red*), respectively; **d** shows the images of GNPs (*left*) and GAL-PEG-GNPs (*right*) detected by TEM

There was no significant difference in appearance between GNPs solution and GAL-PEG-GNPs solution (Fig. 2a). Highly monodisperse GNPs had approximately 20 nm gold cores and average diameters of about 23 nm. In contrast, GAL-PEG-GNPs had dimensions between 13 and 74 nm with a mean size of 34 nm (Fig. 2b). UV–Vis spectroscopy showed that the GNPs and GAL-PEG-GNPs exhibited strong absorption peak at 520 and 523 nm, respectively (Fig. 2c). Further, GNPs and GAL-PEG-GNPs were stained with 2 % phosphotungstic acid. In contrast to GNPs, GAL-PEG-GNPs displayed a thin white ring structure on surface (Fig. 2d), indicating well attachment of GAL on GNPs surface.

### CCK8 assay

**Fig. 3:**
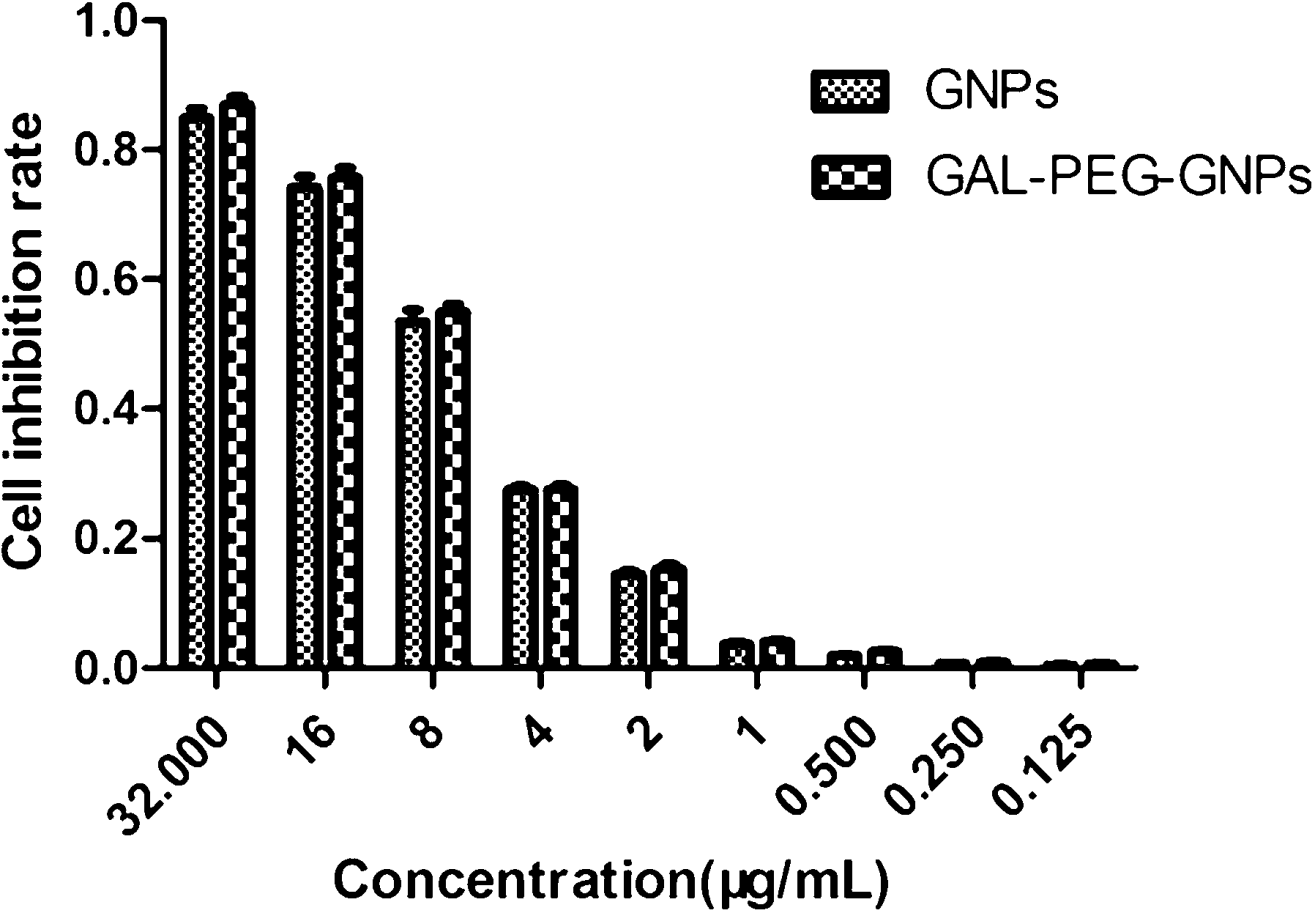
Cytotoxicity of GNPs and GAL-PEG-GNPs at various concentrations in HepG2 cells. The abscissa indicates drug concentration; the ordinate indicates cell inhibition rate. A colorimetric CCK8 assay was performed to measure the cytotoxicity; the measurements follow vender’s protocol

A colorimetric CCK8 assay was performed to measure the cytotoxicity of either GNPs or GAL-PEG-GNPs at various concentrations in cultured HepG2 cells. The results showed that cell viability from both groups decreased with the increasing concentration of nanoparticles. However, there was a significant difference in cell inhibition rate between GNPs-treated cells and GAL-PEG-GNPs-treated cells (*p* < 0.05) (Fig. 3). Further, the IC_50_ value of GNPs and GAL-PEG-GNPs in HepG2 cells was determined to be 5.001 and 4.997 μg/mL, respectively.

### Uptaking GAL-PEG-GNPs by HepG2 Cells

**Fig. 4:**
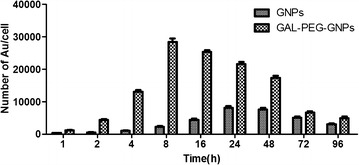
Amount of GNPs and GAL-PEG-GNPs uptaken by HepG2 cells at various timepoints. The Abscissa indicates time; the ordinate indicates numbers of GNPs in one cell. Cells were resuspend in 5 mL of 1× PBS and lysed using 5 mL of Aqua Regia (HNO_3_:HCL = 1:3) solution at RT for 48 h. Concentration of Au in lysates was determined by ICP-MS

**Fig. 5:**
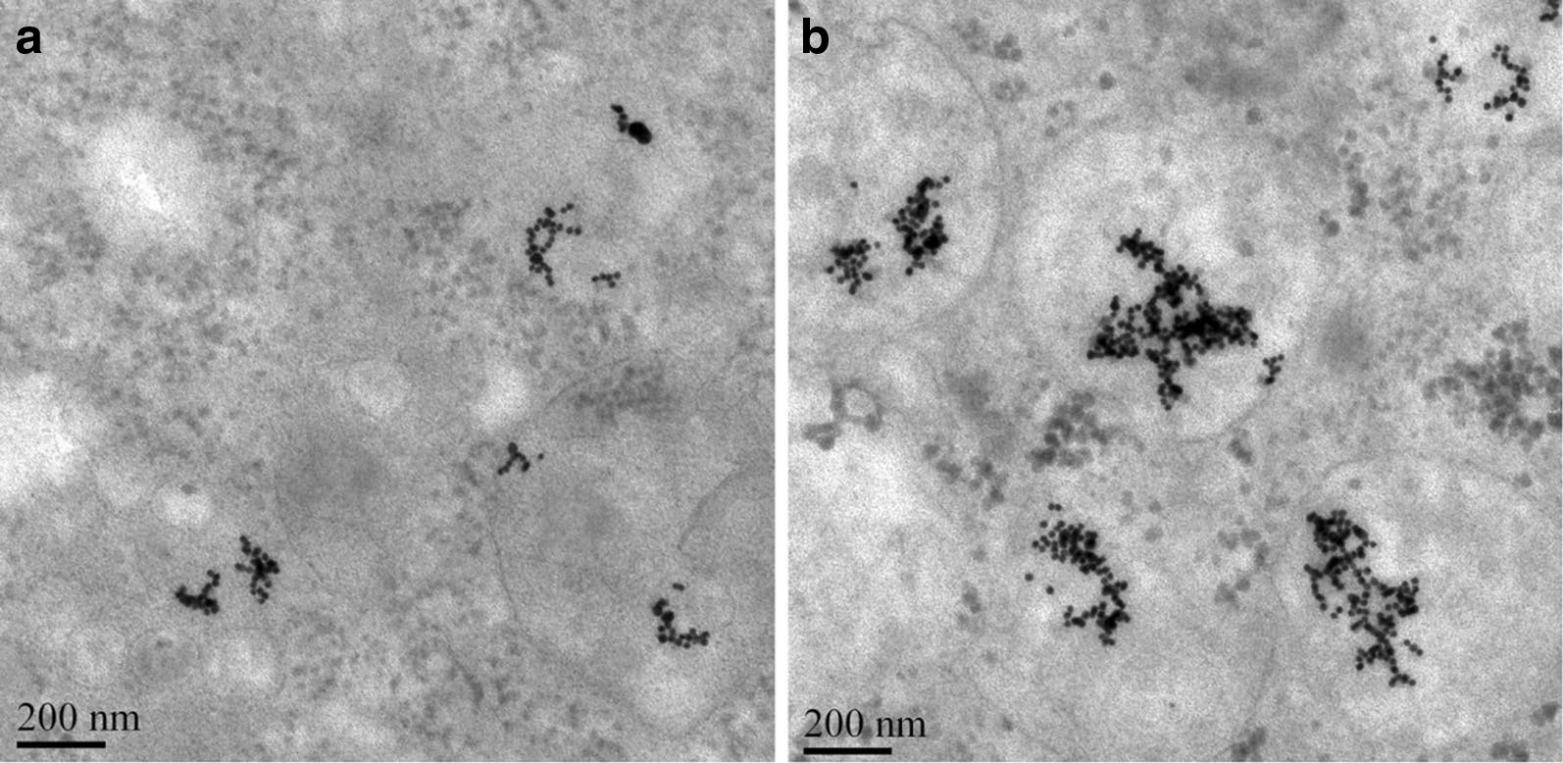
TEM images of GNPs (**a**) and GAL-PEG-GNPs (**b**) uptake in HepG2 cells. Images were taken by FEI Tecnai Spirit transmission electron microscopy (JEM-100CX II, Japan) at 200 kV

The amount of gold uptake was measured by ICP-MS (Fig. 4). No gold was detected in the unexposed control group. In GNPs-treated cells, the gold uptake clearly increased with the incubation time, and the amount of gold reached a peak at 24 h time point. In the subsequent 48–96 h, GNPs were gradually metabolized from cells and thus the amount of GNPs decreased accordingly. In contrast, the amount of GAL-PEG-GNPs uptaken by cells reached a peak at 8 h time point, and approximately three times more gold in cells compared to GNPs-treated cells (*p* < 0.001). The results indicated that GAL-PEG-GNPs could be more quickly and effectively uptaken by HepG2 cells in vitro. TEM images further confirmed that the amount of GAL-PEG-GNPs uptaken in HepG2 cells was much higher than that of GNPs (Fig. 5). Both GNPs and GAL-PEG-GNPs mainly distributed in mitochondria.

### Cell cycle

**Fig. 6:**
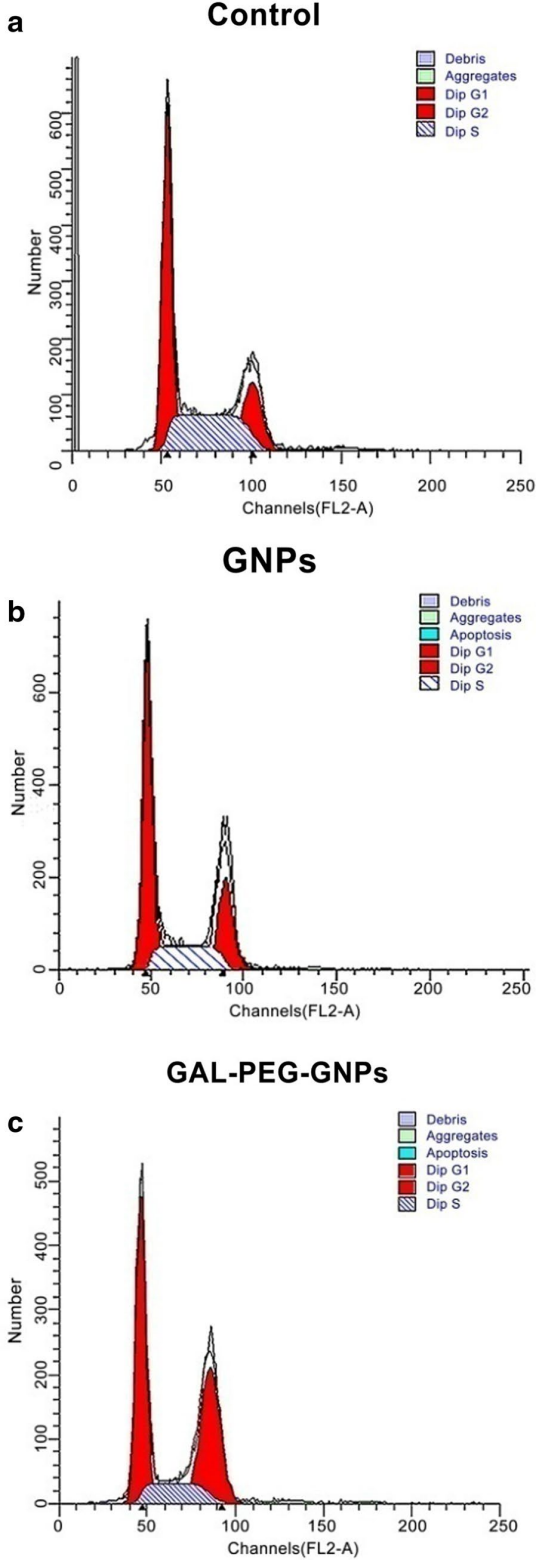
Cell cycle distribution of HepG2 was detected by flow cytometry (n = 3). **a** Control: G0/G1 phase (60.26 ± 2.96) %, S phase (31.65 ± 0.81) %, G2/M phase (8.09 ± 2.2) %. **b** GNPs: G0/G1 phase (55.89 ± 1.7) %, S phase (23.97 ± 0.54) %, G2/M phase (20.26 ± 2.09) %, **c** GAL-PEG-GNPs: G0/G1phase (55.39 ± 1.25) %, S phase (17.67 ± 1.01) %, G2/M phase (27.03 ± 0.7) %. (*p* < 0.01) compared to Control; (*p* < 0.01) compared to Control and GNPs group

After exposure of cells to radiation cells showed varying proportion in S phase, 31.65 % in control group, 23.97 % in GNPs-treat group, and 17.67 % in GAL-PEG-GNPs-treated one, respectively (Fig. 6). In contrast, cell proportion in G2/M phase increased from 8.09 % in control group to 20.26 % in GNPs-treat group and further to 27.03 % in GAL-PEG-GNPs-treated group (*p* < 0.01).

### Clonogenic assay

**Fig. 7:**
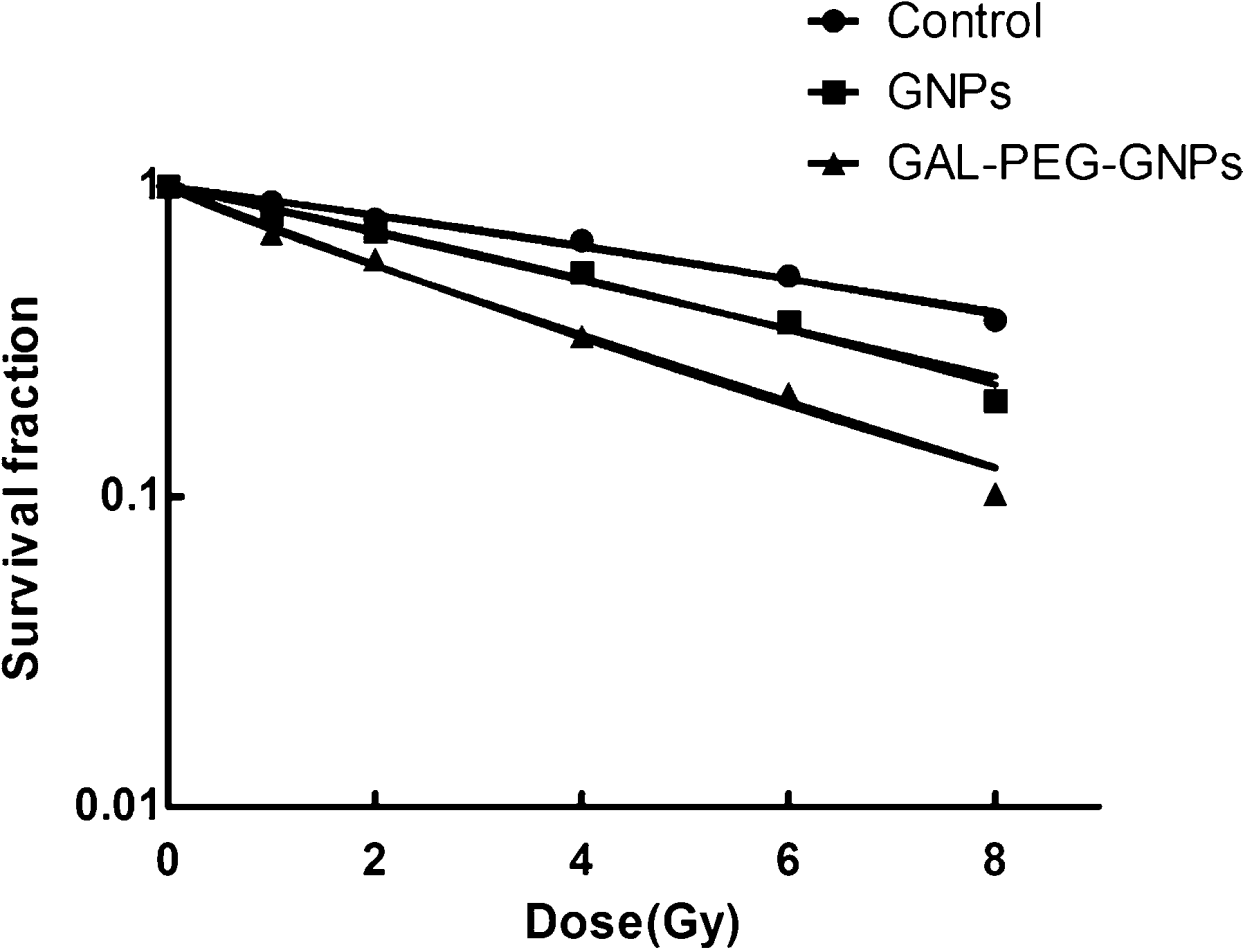
Clonogenic assays demonstrate the radiosensitization effects of GNPs and GAL-PEG-GNPs to HepG2 cells. The cells were exposed to 0 to 8 Gy of 6 MeV X-ray. A dose-dependent clonogenic survival of HepG2 cells. The regression curves were fit to the linear-quadratic model. The SERs of GNPs/X-ray group and GAL-PEG-GNPs X-ray group were 1.46, and 1.95, respectively

The clonogenic survival of HepG2 cells was assessed by treating the cells with X-ray alone or combining X-ray with either GNPs or GAL-PEG-GNPs. The results showed that cell viability from all three groups decreased with increasing radiation dose (Fig. 7). It was noted that cell viability in GAL-PEG-GNPs/X-ray treated group was significantly lower than that of the rest two groups from 1 to 8 Gy (*p* < 0.05), indicating GAL-PEG-GNPs could enhance radiation sensitivity of HepG2 cells to X-ray. According to the fitting cell survival curve, obtained by D0, the SERs of GNPs/X-ray group and GAL-PEG-GNPs/X-ray group were 1.46, and 1.95, respectively.

### Immunofluorescence γ-H2AX Assay

**Fig. 8:**
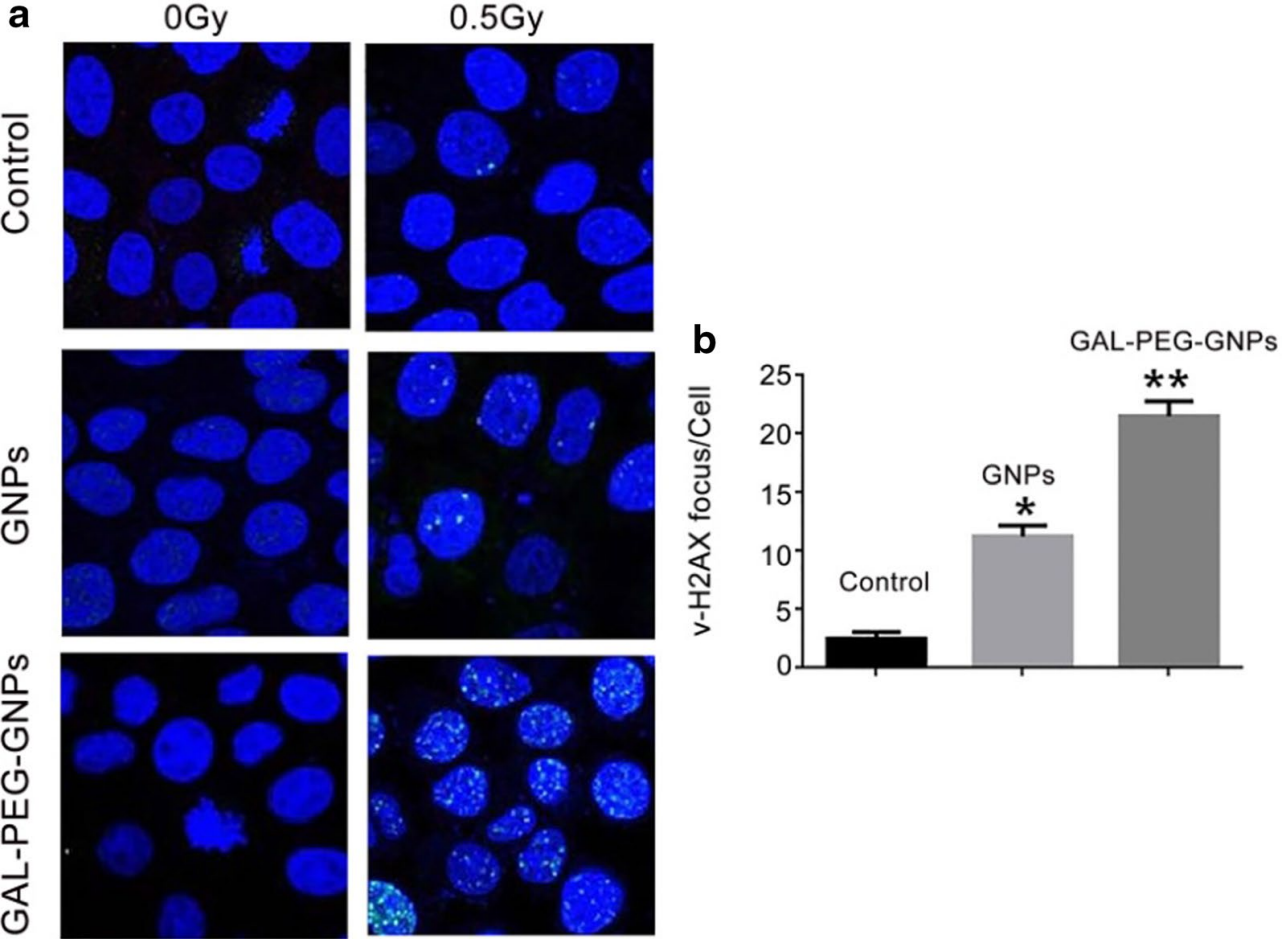
Immage of γH2AX focus in HepG2 cells. **a** Shows the γH2AX focus in HepG2 cells. **b** Sows DSB induction as determined by the γ-H2AX assay was quantified as focus integrated density per unit nucleus area using Image J software. Data represent the mean results from three independent experiments. Compared with the control group, *p < 0.001, compared with GNPs, **p < 0.001

The DNA-DSBs in HepG2 cells exposed to 0.5 Gy X-ray (6 MeV) was measured by the γ-H2AX assay. Untreated cells, GNPs- or GAL-PEG-GNPs-treated cells without radiation exposure did not show DNA-DSBs. In contrast, the formation of γ-H2AX foci in GNPs- and GAL-PEG-GNPs-treated cells with radiation exposure appeared to be 4.5- and 8.6-fold respectively (*p* < 0.001) higher than that of cells with radiation exposure only (Fig. 8).

### Western blot assay

**Fig. 9:**
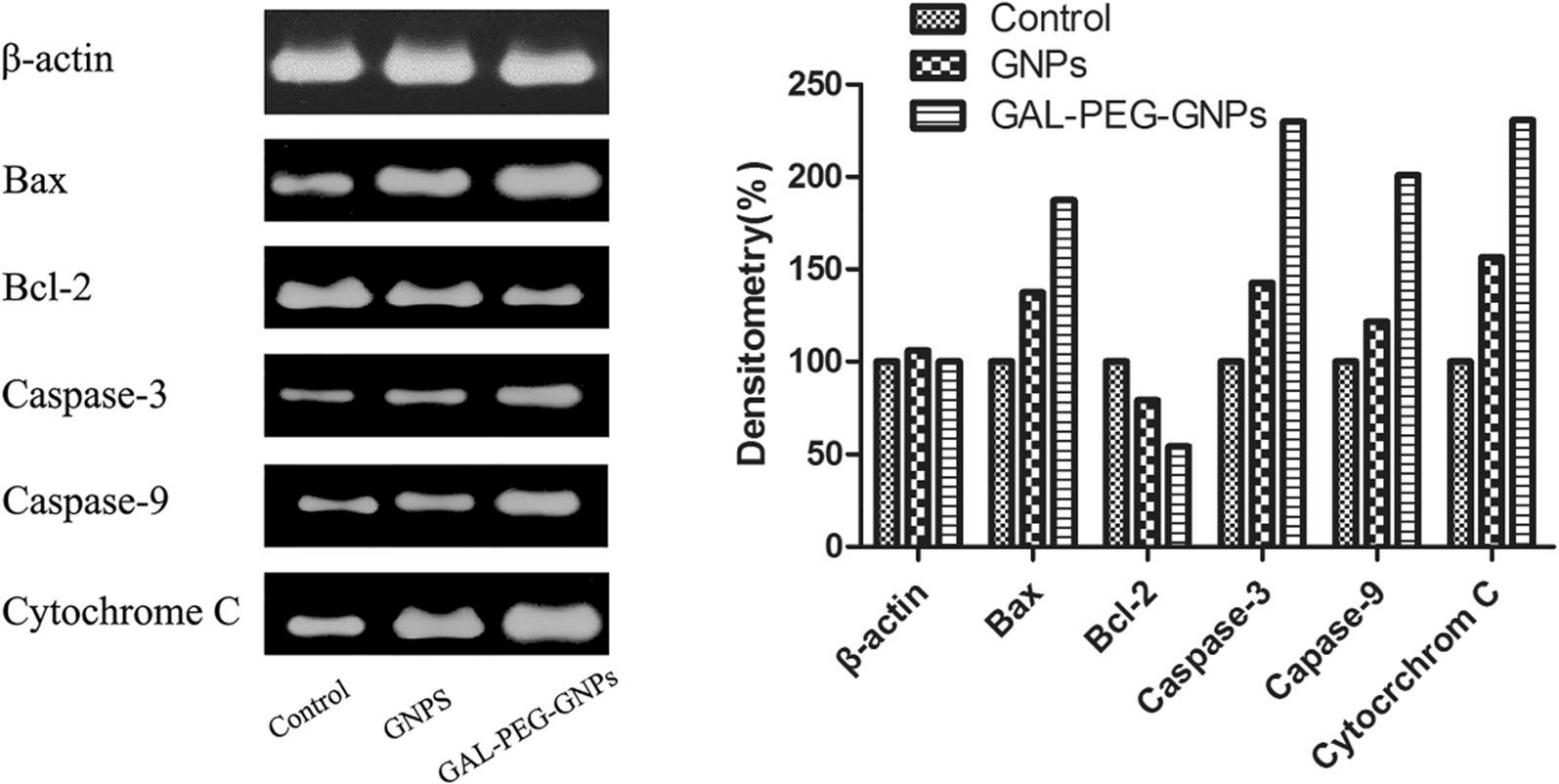
Expression of apoptotic proteins and anti-apoptotic protein in HepG2 cells radiated by 0.5 Gy X-ray

The expressions of Cytochrome C, Bax, caspase-3, caspase-9, and Bcl-2 in cells treated with radiation alone, GNPs/radiation, or GAL-PEG-GNPs/radiation were measured using Western blotting (Fig. 9). The expression of Cytochrome C, Bax, caspase-3, and caspase-9 were upregulated while Bcl-2 expression was downregulated in cells treated with GNPs/radiation or GAL-PEG-GNPs/radiation. These results indicated that gold nanomaterials could induce more expression of intracellular apoptotic molecules and significantly inhibit expression of anti-apoptotic protein in comparison with cells treated with radiation only.

### Expression changes of CAT, SOD and GSH

The levels of three antioxidant proteins, CAT, SOD and GSH that have been used to quantify oxidative stress in cells were measured. Compared with cells treated with radiation, the activity of CAT, SOD, and GSH in the GNPs-treated HepG2 cells with radiation exposure decreased 74.22, 84.13, and 76.47 %, respectively (*p* < 0.01). Meanwhile, the activity of CAT, SOD and GSH in GAL-PEG-GNPs-treated cells with radiation exposure decreased 51.56, 61.16, and 54.90 %, respectively (*p* < 0.01). There was a significant difference in expression of these proteins between two experimental groups (*p* < 0.01) as well (Table 1).

**Table 1:** CAT/SOD/GSH changes in HepG2 cells detected by the Microplate reader (mean ± SD)

Group	CAT (U/g)	SOD (U/mg)	GSH (μmol/g)
Control	128 ± 4.35	466 ± 5.19	51 ± 2.08
GNPs	95 ± 7.09	392 ± 8.54	39 ± 2
GAL-PEG-GNPs	66 ± 6.56	285 ± 7.94	28 ± 1.15

## Discussion

Nanoparticles can permeate leaky angiogenic endothelium providing some tumor specificity. Additionally, blood clearance of nanoparticles is slower than small molecules, such as iodine contrast media, and thus nanoparticles can stay in the blood for hours [23]. Moreover, drugs can be bound to the nanoparticles or be entrapped inside the nanoparticles, allowing flexible and multifunctional therapy [24]. Nevertheless, nanoparticles have the potential to revolutionize cancer diagnosis and therapy [25]. Among various nanomaterials, particularly GNPs are very attractive. They can be safely administered with minimal inflammatory activation and few side effects. GNPs are very convenient to realize a variety of surface functionalization with various biological molecules to help improve stability, drug-carrying, and tumor-targeting [12]. Furthermore, gold allows high absorption and enhancement of ionizing radiation, as well as superior X-ray attenuation for biomedical imaging application. Moreover, other physical properties including surface plasmon resonance and Raman scattering activity have been exploited in fluorescent imaging, photothermal therapy, and many other applications [26, 27]. At KeV or MeV radiation energies, GNPs can selectively improve radiosensitivity of tumor cells leading to increased cell killing and have been trialed on various cancer cell lines and animals [28–31]. Theoretically, the dose enhancement achieved by GNPs could be more than 200 % [16]. Though the mechanisms are still unclear, radiosensitization is generally attributed to increasing photo absorption of high-Z elements, and the resulting transfer of a larger portion of primary ionizing photon energy to tumor tissue.

Our previous results showed that bare GNPs could improve the radiosensitivity of HepG2 cells, elevate DNA damage levels, and induce cancer cells apoptosis in vitro [14]. To deliver a high dose of GNPs directly to malignant cells, and to specifically radiosensitize them while minimizing side-effects, we further graft GAL targeting ASGPR for delivering GNPs to HepG2 cells in vitro. First of all, GNPs were prepared by sodium citrate reduction followed by surface functionalization with SH-PEG-NH_2_ molecules and surface deactivation using SH-PEG molecules. The grafted amine groups were used to covalently immobilize GAL.

The size of GNPs for liver cancer therapy should not exceed 200 nm, since the size of liver sinusoidal fenestrations have 200 nm effective size limit. On the other hand, GNPs may experience rapid renal clearance if they are smaller than 10 nm in diameter. Size is also an influencing radiation sensitivity parameter, and large sized GNPs have superior dose enhancement factor [32, 33]. Bergen et al. tested the efficacy of GNP size ranging from 50 to 150 nm and concluded that GNPs in 50-nm diameter could have the highest radio enhancement factor (1.43 at 220 keV) and also have the highest cellular uptake [16]. However, a recent report showed that GNPs in 18-nm diameter have more cell internalization [34]. Though the optimal size of GNPs is inconclusive so far, there is a tradeoff between effective dose enhancement of large GNPs and the effective clearance of small ones. This remains a hurdle for their application in cancer imaging and therapy. The average size of our GNPs and GAL-PEG-GNPs is 23 and 34 nm, respectively. Therefore, our nanoparticles prepared in this study are in principle suitable, in terms of size for radiosensitization applications, exploiting the enhanced permeability and retention effect and avoiding renal clearance. The larger size and peak shift of GAL-PEG-GNPs are attributed to surface PEGylation and GAL grafting. PEGylation can further reduce the reticuloendothelial system uptake and thus significantly increase circulation time. In addition, PEGylation decreases aggregation of GNPs owing to passivated surfaces and increase solubility in buffer and serum due to hydrophilic ethylene glycol repeats. It was reported that PEGylated nanoparticles show higher tumor accumulation versus background.

Both GNPs and GAL-PEG-GNPs at various concentrations showed cytotoxicity, and their IC_50_ values are 5.001 and 4.997 μg/mL, respectively. Compared to the published data of GNPs cytotoxicity [35], our GNPs are relatively less cytotoxic. In this study, we merely used 1/5 concentration (1.0 μg/mL) to minimize the cytotoxicity on HepG2 cells, and thus such low level of cytotoxicity can be neglected.

ICP-MS analysis revealed that the amount of GAL-PEG-GNPs endocytosed by HepG2 cells was approximately three times more than that of GNPs dose, suggesting GAL significantly increased the gold uptake. Although both internalizing and noninternalizing epitopes on cell membrane can be targeted, if ligands bind to noninternalizing ones, drugs or nanoparticles may accumulate around the cells and enter into cells either by passive diffusion or normal transport mechanisms. In contrast, when GAL bind to internalizing epitopes, like ASGPR, the binding triggers ASGPR-mediated uptake and hence GAL-PEG-GNPs uptake is thus more effective. TEM images showed that GNPs were mainly distributed in mitochondrion. Few reports have demonstrated a role for reactive oxygen free radicals (ROS) or the involvement of mitochondria as mechanism of GNPs radiosensitization [36, 37]. In this study, we confirmed that elevated levels of DNA damage which may be a direct result of impaired mitochondrial function manifested by increased oxidation and loss of membrane potential, resulting in HepG2 cells proliferation inhibition. It was further confirmed by clonogenic analysis. After exposure of cells to various radiation doses (1–8 Gy zone), clone quantities in GAL-PEG-GNPs-treated group were significantly less than that in GNPs-treated and control groups mainly due to more amount of gold uptake.

The cell cycle analysis showed that cells were propelled to enter G2/M phase. GAL-PEG-GNPs induced approximately 27 % of cells in the G2/M phase; the percentage was more than that of GNPs induced. G2/M phase is the most radiosensitive phase of a cell cycle, and thus more accumulation in G2/M phase can enhance the radiation sensitivity. It was reported that GNPs can trigger activation of the CDK kinases, leading cancer cells to accumulate in the G2/M phase [37, 38]. Additionally, p53 protein may mediated G1 arrest and p53-independent G2/M phase arrest over 2–10 h after radiation [39]. Moreover, cell cycle kinetics can be changed by GNPs after radiation. Previous reports demonstrated that GNP radiosensitization can increase sub G1 population or accumulate cells in G2/M. Additionally, surface functionalization of GNP using a range of different biological moieties not only enables them maintain stability and bio-compatibility but also leads to accumulation of GNPs in tumors compared to healthy tissues [40]. Thus, it is likely to provide significant therapeutic advantage and may potential benefit personalized medicine.

γ-H2AX assay results showed a significantly higher incidence of DNA-DSBs in GAL-PEG-GNPs-treated group as compared to that in GNPs-treated or control group. Decreased clonogenic survival of HepG2 cells and increased DNA-DSBs demonstrated that GAL-PEG-GNPs achieved better radiotherapy sensitization effect compared to bare GNPs.

Significantly upregulated expression of Cytochrome C, Bax, Caspase-3, and Caspase-9 and downregulated anti-apoptotic protein Bcl-2 were found in GAL-PEG-GNPs-treated cells after radiation. We found antioxidant enzymes including CAT, SOD, and GSH were significantly reduced indicating the increase of free radicals in HepG2 cells. The release of ROS can cause mitochondrial membrane depolarization and oxidization, and subsequently attack intercellular macromolecules, such as DNA, RNA, proteins, leading to above mentioned changes [31, 32]. The cytochrome C, which was released from mitochondria to cytoplasm activated Caspase-9; Caspases-9 further activated Caspase-3 and others [33, 34]. Caspase-3 caused chromatin pyknosis, DNA fragmentation, and DNA fracture leading to cell apoptosis [35]. In contrast, the mechanisms of upregulated Bax and downregulated Bcl-2 through GNPs-mediated radiation are not clear yet, and thus should be further investigated in the future.

## Conclusions

In this study, we successfully prepared GAL-PEG-GNPs as a novel radiosensitizer,found our novel nanoparticles could significantly improve the efficacy of radiotherapy in vitro, and briefly discussed the mechanism of radiosensitization. Given the wide range of methods available to further modify these nanoparticles, in ongoing work we are also exploring the use of cell-specific internalizing aptamers (i.e. anti-EGFR aptamers or anti-EpCAM aptamers) grafted PEG-GNPs that can more effectively be taken up by cancer cells overexpressing a cognate receptor, and the dual delivery of chemotherapeutic agents and GNPs to achieve a synergistic therapeutic outcome in vivo. These vehicles may be able to circumvent biological barriers, such as the blood–brain barrier, thus resulting in efficient localization of various agents at the target sites.

## Methods

### Cell culture

The HepG2 cells were cultured in DMEM (GIBCO, Grand Island, USA) supplemented with 10 % heat-inactivated fetal calf serum (FCS, GIBCO, Grand Island, USA) and 1 % penicillin–streptomycin (GIBCO, Grand Island, USA) at 37 °C under a humidified atmosphere containing 5 % CO_2_ and maintained in an exponential growth state. Cells were passaged once every 3 days by using 0.25 % trypsin.

### Preparation of GAL-PEG-GNPs

Synthesis of GNPs in 20 nm diameter was done with sodium citrate reduction method. The preparation method can be found elsewhere [21]. To graft amine groups onto bare GNPs surfaces, 0.1 mL of GNPs at 0.1 mg/L (pH 7.5) were well mixed with excessive amount of thiol-PEG-amino (5000 Da, Seebio, ShangHai, China) and incubated at 4 °C overnight. The following thiol-PEG (5000 Da, Seebio, ShangHai, China) surface modification was performed to thoroughly replace the residual citrate groups. The products were purified and buffer exchanged into 1× PBS (pH 7.5) using ultrafiltration. 100 μL of *N*-hydroxysulfosuccinimide (sulfo-NHS) and carbodiimide (EDC) (5:1, pH 5.0) were incubated with 20 μg of GAL for 6 h at 37 °C to active carboxyl groups of GAL. At pH 7.2, 20 μL of PEGylated GNPs at 0.1 mg/L were added and incubated with the mixture at 37 °C for 2 h to finally produce GAL-PEG-GNPs (Fig. 1). The products were concentrated, filtered and stored at 4 °C.

### Characterization of GAL-PEG-GNPs

Dynamic light scattering (DLS) was used to determine the size of GNPs. The DLS and Z-potential experiments were performed on a Malvern Zetasizer Nano ZS (Malvern Instruments, Southborough, Massachusetts). Samples filtered followed by equilibration (typically 5 min) in a quartz cuvette to 37 °C. The software was arranged with the specific parameters of refractive index and absorption coefficient of the material and the viscosity of the solvent. DLS allows determination of hydrodynamic diameter of colloidal particles and conjugates, that is the diameter of sphere with the same Brownian motion as the analyzed particle. The concentration of GNPs and GAL-PEG-GNPs were determined by using inductively couple plasma mass spectrometry (ICP-MS). UV–visible adsorption spectrum of GNPs or GAL-PEG-GNPs was acquired over the wavelength range from 250 to 800 nm with UV-2450 spectrophotometer (Tianjin Gangdong Sci. & Tech. Development CO. LTD, China) using quartz cuvettes with an optical path length of 0.5 cm at room temperature (RT). The size and morphology of GAL-PEG-GNPs are analyzed by FEI Tecnai Spirit transmission electron microscopy (TEM) (JEM-100CX II, Japan) at 200 kV using AnalySIS software (Soft Imaging Systems).

### CCK8 assay

The CCK8 assays were performed as instructed by the manufacturer to assess cell viability. Briefly, the cells were seeded in 96-well plates (approximately 3000 cells/well). GNPs and GAL-PEG-GNPs were diluted to various concentrations in 1× PBS (pH 7.4), then added into the wells. After 24 h incubation, 20 μL CCK8 (keygentec company, Nanjing, Jiangsu, China) was added to each well for 4 h. Optical density (OD) was measured at 490 nm using a Microplate reader (BioRad, DG3022, USA) and the proliferation index was calculated as experimental OD value/control OD value. Estimate value of 50 % inhibition concentration (IC_50_).

### ICP-MS assay

The assay was performed in triplicate. A total of 1 × 10^6^ HepG2 cells were seeded onto a culture dish (dia. 15 mm) and cultured for 24 h. When the cells reached a 70 % confluence, cells were exposed to GNPs (1/5 IC_50_ = 1.0 μg/mL) and GAL-PEG-GNPs (1/5 IC_50_ = 1.0 μg/mL) at 37 °C for 1, 2, 4, 8, 12, 24, 48, 72, 96 h, respectively. Cells were washed with 1× PBS twice, detached with 0.25 % trypsin, and suspend in 5 mL of 1× PBS. 5 mL of Aqua Regia (HNO_3_:HCL = 1:3) solution was added to cell suspension to fully lyse cells at RT for 48 h. Concentration of Au in lysates was determined by ICP-MS, the total number of nanoparticles and cells were recorded as *n* and *N*, respectively. Thus, the number of nanoparticles contained in each cell calculated as n/N.

### Transmission electron microscopy

A total of 1 × 10^6^ HepG2 cells were seed onto a culture dish (dia. 15 mm) and cultured for 24 h. After another 24 h of incubation with GNPs or GAL-PEG-GNPs, cells were fixed by 4 % paraformaldehyde/2.5 % glutaraldehyde in PBS (0.7 mL) for 3 h. The cells were next rinsed with PBS and post-fixed using 1 % aqueous solution of OsO_4_ (0.5 mL) for 1 h. Subsequently, the cells were washed with DI water, 30 % ethanol solution and stained with 0.5 % uranyl acetate (0.5 mL, in 30 % ethanol) for 1 h. Cells were then gradually dehydrated using a series of ethanol solutions (30, 60, 70, 80, and 100 %) and embedded in epoxy resin. The resin was polymerized at 60 °C for 48 h. Ultra-thin sections (70–100 nm) were cut using a diamond knife on a Leica Ultramicrotome and mounted on Formvarcoated copper grids. The sections were then post-stained with 5 % uranyl acetate in 50 % ethanol and 2 % aqueous lead citrate solution and imaged with TEM at 200 kV.

### Cells cycle assay

The cells exposed to 0.25 Gy X-ray (6 MeV, Medical linear accelerator, Germany Siemens Primus Company) after incubating with GNPs (1.0 μg/mL) or GAL-PEG-GNPs (1.0 μg/mL) for 24 h and then were harvested using 0.25 % trypsin with 1 mM EDTA solution and fixed for 12 h in 70 % ethanol at 4 °C. The fixed cells were then centrifuged at 3000 rpm for 15 min to remove the ethanol thoroughly. The cells were then washed twice with 3 mL of PBS, resuspended in 1 mL of PI (Sigema, USA) staining solution, and incubated for 15 min at RT. The staining solution consisted of 20 mg/mL PI and 0.2 mg/mL RNase in PBS. The samples were subsequently analyzed using a BD FACS CantoII instrument (BD Biosciences, USA). Twenty thousand events were collected from each sample. The percentages of cells in the G0/G1, S, and G2/M phases of the cell cycle were determined using the ModFit software (BD, USA).

### Clonogenic assay

The radiosensitization of GAL-PEG-GNPs or GNPs to HepG2 cells were assessed by the clonogenic assay. Different number of HepG2 cells (100, 300, 1000, 5000, 10,000) were plated in 6-well and incubated with GNPs (1.0 μg/mL) or GAL-PEG-GNP (1.0 μg/mL) for 24 h, then irradiated with 0, 1, 2, 4, 6, 8 Gy X-ray and incubated for 9–14 days. The colonies were fixed with methanol and stained with 0.4 % crystal violet. Finally, the plates were inspected by microscopy and the number of the colonies was counted. Each assay was made in triplicate and only colonies containing at least 50 cells were counted. The sensitizer enhancement ratio (SER) was calculated as the radiation dose needed for radiation alone divided by the dose needed for various concentrations of nanoparticles plus radiation at a survival fraction of 37 % (D_0_ in radiobiology).

### DNA damage immunofluorescence microscopy

The ability of GAL-PEG-GNPs or GNPs to enhance DNA double-strand breaks (DSBs) in HepG2 cells exposed to 0.5 Gy X-ray was evaluated using the *γ*-H2AX assay. This assay detects the phosphorylation of histone-H2AX at serine-139 (*γ*-H2AX), which is visualized as discrete nuclear foci by laser confocal microscopy using *γ*-H2AX-specific antibodies.

Cells were cultured (6 × 10^4^ cells/well) on 24-well plates overnight at 37 °C in medium. After 24 h, the culture medium was replaced with 300 mL of fresh medium or medium containing GAL-PEG-GNPs or GNPs, respectively, and incubated overnight at 37 °C. The treated cells were then exposed to 0.5 Gy of X-radiation using an X-ray source, operating at 6 MeV. Cells were subsequently fixed using 4 % paraformaldehyde (Sigma-Aldrich, USA), permeabilized with 0.5 % Nonidet P40 (Sigma-Aldrich, USA) in PBS for 15 min, and blocked in 2 % bovine serum albumin (BSA, Sigma-Aldrich, USA) for 1 h at RT. Cells were then incubated with anti-*γ*-H2AX mouse monoclonal IgG1 (Upstate Biotechnology, Billerica, MA, USA) at a 1:800 dilution in 3 % BSA-PBS overnight at 48 °C and then with anti-mouse IgG (H + L) (Invitrogen Molecular Probes, Carlsbad, CA, USA) at 1:500 dilution for 45 min at RT. Cover glasses were mounted on microscope slides (25 × 75 × 1 mm, Fisherbrand, Fisher Scientific Co.) The edges of cover glasses were sealed with clear nail polish. From the step using the secondary antibody onwards, all procedures were performed in the dark. Cover glasses were wrapped in aluminum foil and stored at 4 °C for later image acquisition. Images of *γ*-H2AX foci and nuclei were acquired with a Confocal Microscope Images were taken with an inverted laser confocal microscope (Zeiss510, Germany). Excitation was at 364 or 488 nm for visualization of DAPI or AlexaFluor-488 with emission filters of 385–470 nm or 505–550 nm, respectively. The number of *γ*-H2AX foci present in each cell was quantified with Image J software (version 1.36b, National Institutes of Health) using customized macros recently developed by our group.

### Western blot analysis

Cellular proteins were extracted, quantified, and subjected to sodium dodecylsulfate polyacrylamide gel electrophoresis (SDS-PAGE, keygentec company, KGP113). Proteins samples were then blotted onto a nitrocellulose membrane. After incubation with a blocking buffer, the membranes were incubated for 2 h at RT with each primary antibody at the appropriate dilution, as recommended by the supplier. Antibodies included Bax, Bcl-2, Caspase-3, Caspase-9, and Cytochrome C. After washing, the membranes were subsequently incubated for 1 h at RT in Goat Anti-mouse Immunoglobulin G (IgG, keygentec company, KGAA36) followed by the use of enhanced chemiluminescence kits. β-actin was used as an internal control.

### Expression of CAT, SOD, and T-GSH in HepG2 cells^14^

Catalase (CAT) activity was estimated by the method of Aebi. Activity of the enzyme superoxide dismutase (SOD) was measured by nitro blue tetrazolium reduction method of McCord and Fridovich. The level of glutathione (GSH) was assayed by the method of Moron et al. based on the reaction with dithiobis(2-nitrobenzoic acid). The method measured the rate of decomposition of hydrogen peroxide (H_2_O_2_) at 240 nm.

### Statistical analysis

In the statistical analysis, differences between the treated and control groups were compared using Student’s *t* tests, with the differences at the *P* < 0.05 level considered to be statistically significant.
